# Orthologues of the human protein histidine methyltransferase METTL9 display distinct substrate specificities

**DOI:** 10.1016/j.jbc.2025.110318

**Published:** 2025-05-30

**Authors:** Lisa Schroer, Sara Weirich, Marta Hammerstad, Hans-Petter Hersleth, Ida Andrietta Grønsberg, Lars Hagen, Geir Slupphaug, Jedrzej Mieczyslaw Malecki, Albert Jeltsch, Pål Ø. Falnes, Erna Davydova

**Affiliations:** 1 Section for Biochemistry and Molecular Biology, Department of Biosciences, University of Oslo, Oslo, Norway; 2 Department of Molecular Biochemistry, Institute of Biochemistry, University of Stuttgart, Stuttgart, Germany; 3 Faculty of Medicine and Health, Department of Clinical and Molecular Medicine, Norwegian University of Science and Technology, Trondheim, Norway; 4 Clinic of Laboratory Medicine, St Olav's Hospital, Trondheim, Norway; 5 Proteomics and Modomics Experimental Core, PROMEC, at NTNU and the Central Norway Regional Health Authority, Stjørdal, Norway; 6 CRESCO - Centre for Embryology and Healthy Development, University of Oslo and Oslo University Hospital, Oslo, Norway

**Keywords:** histidine, protein methylation, protein structure, protein evolution, protein motif, peptide array, histidine methyltransferase, methylhistidine

## Abstract

The human (*Homo sapiens*; Hs) methyltransferase (MTase) METTL9 is the first enzyme shown to generate 1-methylhistidine (π-methylhistidine) in proteins. METTL9 preferentially methylates an alternating histidine (HxH) motif, where “x” is a small, uncharged amino acid, and multiple substrates have been identified. Putative METTL9 orthologues are found in most eukaryotes, and we have here investigated the activity of such enzymes from several species, representing all five eukaryotic supergroups. The majority of the tested enzymes demonstrated *in vitro* MTase activity on the prototype HsMETTL9 substrates ARMC6 and DNAJB12. We also detected protein methylation activity of the *Caenorhabditis elegans* METTL9 which had previously been suggested to be a DNA MTase. However, METTL9 from the fruit fly *(Drosophila melanogaster*; Dm) and the picoplankton *Ostreococcus tauri* (Ot) displayed distinct substrate specificities, differing from each other and from that of HsMETTL9. These differences were observed when recombinant proteins and short peptides were used as METTL9 substrates. To further analyze substrate specificity, we used peptide arrays to systematically replace the “x” residue and the residues flanking the HxH motif in a substrate peptide. This revealed varying degrees of tolerance among the METTL9 orthologues (Hs > Dm > Ot) for substitutions at these positions. Our results show that the METTL9 orthologues, although requiring an HxH target site, have evolved different substrate specificities, likely due to differing biologically relevant substrates in the respective organisms. Furthermore, we solved the X-ray structure of OtMETTL9, revealing several differences from the previously published HsMETTL9 structures that may explain its distinct substrate specificity.

Methylhistidine is an emerging post-translational modification recently identified in hundreds of human proteins (1, 2). So far, only four human histidine-specific methyltransferases (MTases) have been identified, each using *S*-adenosyl-L-methionine (SAM) as a methyl donor. Two of these enzymes generate 1-methylhistidine (1MH; also referred to as π-MH), and two generate 3-methylhistidine (3MH; also referred to as τ-MH). The 3MH MTases, SETD3 and METTL18, methylate a single protein substrate (3, 4, 5), whereas the 1MH MTases, CARNMT1 and METTL9, methylate a large number of proteins, each generating roughly half of the total 1MH content in the mammalian proteome (6, 7).

METTL9 specifically introduces 1MH at histidine-x-histidine (HxH) motifs, where x is preferentially a small residue such as A, N, G, S, T, or C. There are also several sequence constraints on the residues flanking the motif, such as low tolerance for P or V at either side, and for I and E as the residue immediately preceding the motif (7, 8). There are more than 2000 instances of such HxH motifs in the human proteome, so the potential extent of METTL9-mediated methylation is vast (7, 9). Established *in vivo* substrates of METTL9 include the immunomodulatory protein S100A9, zinc transporters of the ZIP/SLC39A and ZNT/SLC30A families, the mitochondrial Complex I subunit NDUFB3, the chaperone DNAJB12, and the armadillo repeat protein ARMC6 (7, 8, 10), but likely many more substrates remain to be identified.

METTL9 has been associated with several diseases, such as hepatocellular carcinoma, scirrhous gastric cancer, HIV, and bacterial infection (11, 12, 13, 14, 15). As an enzyme with many different substrates, it likely exerts its various effects through methylation of distinct subsets of these. For example, METTL9-mediated methylation of S100A9 suppresses anti-*Staphylococcus aureus* activity in mouse neutrophils, and methylation of NDUFB3 was suggested to affect cell migration and invasion in metastatic scirrhous gastric cancer, whereas methylation of zinc transporters has been linked to progression of hepatocellular carcinoma (12, 13, 15). These findings suggest a potential role for inhibitors of METTL9 as therapeutic drugs. A deeper understanding of METTL9's substrate recognition and catalytic mechanism is therefore critical, with the ultimate goal of selectively modulating its activity toward one subset of its methylation targets, while leaving the others unaffected.

METTL9-like protein sequences are present throughout the eukaryotic domain, with the notable absence from land plants and fungi (7). In this study, we have examined the MTase activity of nine METTL9 orthologues spanning all five eukaryotic supergroups. We found distinct substrate specificities among such enzymes from *Drosophila melanogaster* (DmMETTL9) and *Ostreococcus tauri* (OtMETTL9), which differed from each other and from HsMETTL9. While HsMETTL9 methylates both ARMC6 and DNAJB12, DmMETTL9 displayed *in vitro* activity exclusively on ARMC6, whereas OtMETTL9 only methylated DNAJB12. We also investigated methylation of seven different peptides and performed a peptide array replacement analysis of the HxH motif and adjacent residues, using a previously unexamined peptide substrate from MYO18A, which is a target of all three orthologues. We observed that Ot- and DmMETTL9 displayed in general stricter sequence requirements for these residues, as compared to HsMETTL9. We furthermore demonstrate that OtMETTL9 generates 1MH on DNAJB12, in line with the activity of HsMETTL9. Finally, we solved the X-ray crystal structure of OtMETTL9, revealing similarities to the previously published HsMETTL9 structures (16, 17) as well as several differences that could contribute to its distinct substrate preference. These findings provide new insights into the evolution and functional diversity of METTL9 enzymes across eukaryotes.

## Results & discussion

### Selected METTL9 proteins and their sequence features

**Figure 1:**
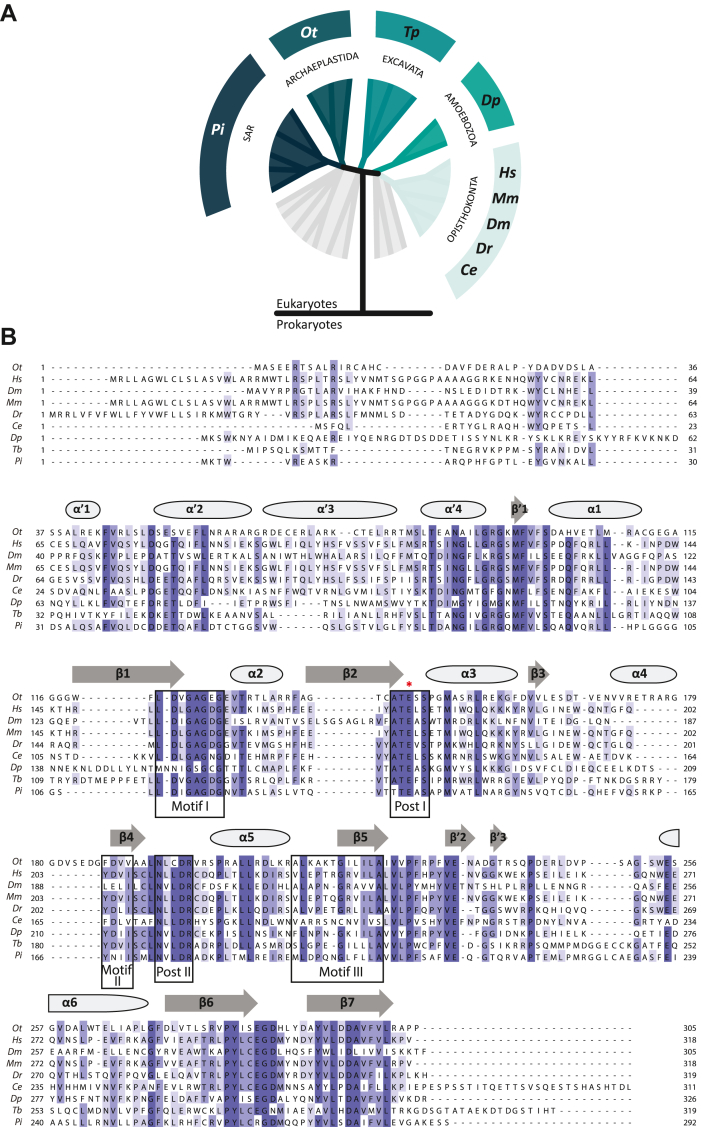
**The METTL9 orthologues selected for this study represent all five eukaryotic supergroups.***A*, evolutionary distribution of the nine METTL9 orthologues selected for this study. An unrooted phylogenetic tree is used to illustrate to which eukaryotic supergroup the respective selected METTL9 orthologues belong. From each supergroup at least one orthologue was chosen. The nine orthologues are from: *Homo sapiens* (Hs), *Mus musculus* (Mm), *Drosophila melanogaster* (Dm), *Danio rerio* (Dr), *Caenorhabditis elegans* (Ce), *Dictyostelium purpureum* (Dp), *Trypanosoma brucei* (Tb), *Ostreococcus tauri* (Ot), and *Phytophthora infestans* (Pi). SAR = Stramenopiles, Alveolata, and Rhizaria. Figure adapted from (18). *B*, sequence alignment of the nine METTL9 orthologues. Secondary structure was assigned from OtMETTL9 structure and is depicted as *ellipses* (α-helices) and *arrows* (β-strands). Hallmark 7BS MTase motifs I-III and Post I-II are labelled and boxed. The β-strands of the core 7BS fold are marked β1-7. The glutamic acid residue mutated in the E174A HsMETTL9, E162A DmMETTL9, E145A OtMETTL9, and E136A CeMETTL9 mutants, which were used as inactive negative controls, is marked with a *red asterisk*.

For this study, we selected nine putative METTL9 orthologues present in representative organisms from all five eukaryotic supergroups, according to the classification of eukaryotes (18). Besides human METTL9 (*Homo sapiens*; Hs; Q9H1A3-1), four orthologues from the Opisthokonta supergroup were included, *i.e*., from the classic model organisms mouse (*Mus musculus;* Mm; Q9EPL4), zebrafish (*Danio rerio*; Dr; F1R335), fruit fly (*D. melanogaster*; Dm; Q9W1H1), and roundworm (*Caenorhabditis elegans*; Ce; Q22123). The remaining four supergroups were represented by one organism each, *i.e.*, Amoebozoa by the slime mold *Dictyostelium purpureum* (Dp; F0ZIA5), Excavata by the protozoan *Trypanosoma brucei* (Tb; Q584Q2), the SAR (Stramenopiles, Alveolata, and Rhizaria) supergroup by the water mold *Phytophthora infestans* (Pi; D0NQJ8), and Archaeplastida by the smallest free-living photosynthetic eukaryote known to date, the single-cell algae *O. tauri* (Ot; Q01C52) (Fig. 1*A*).

METTL9 belongs to the so-called seven β-strand (7BS) MTase family, also referred to as Class I or Rossmann-like MTases. This family includes more than a hundred MTases in humans, which, collectively, target a wide range of substrates, including proteins, DNA, RNA, and various small molecules (19). The 7BS MTases share a three-dimensional fold consisting of alternating β-strands and α-helices, with the β-strands forming a characteristic twisted β-sheet flanked by the α-helices, yielding a three-layered αβα-sandwich structure. Several hallmark sequence motifs are located at distinct locations within the 7BS structure. “Motif I” and “Post I” contain residues important for binding the co-substrate SAM, whereas “Motif II” and “Motif III” are more variable and may be absent in some 7BS MTases. The combination of these motifs and their relative spacing has been successfully used to identify novel 7BS MTases through database searches (20, 21). In addition, the “Post II” motif, which is involved in substrate recognition, is usually highly conserved between MTase orthologues, as well as between paralogues that target similar substrates (19, 22). A sequence alignment of the nine METTL9 orthologues (Fig. 1*B*) revealed a high degree of conservation across these canonical motifs. However, the Ce and Dp orthologues diverged more substantially from the others, with DpMETTL9 deviating in Motif I, and CeMETTL9 in Motif III and surrounding sequences (Fig. 1*B*).

### METTL9 orthologues exhibit different specificities on recombinant protein substrates

**Figure 2:**
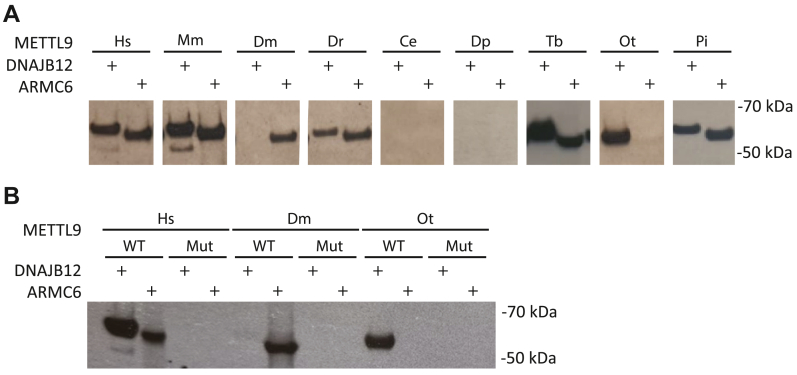
**METTL9 orthologues show distinct *in vitro* methylation preferences.***A*, *in vitro* methylation activity of wild type (WT) METTL9 orthologues on recombinant ARMC6 and DNAJB12. *B*, active site mutations (Mut) abolish the activity of METTL9 orthologues on recombinant ARMC6 and DNAJB12. The mutants are HsMETTL9 E174A, DmMETTL9 E162A and OtMETTL9 E145A.

To determine whether the putative METTL9 orthologues from different species are functional histidine MTases, we investigated the ability of the corresponding recombinant enzymes to methylate the proteins DNAJB12 and ARMC6, two previously established substrates of HsMETTL9 (7). Recombinant METTL9 enzymes, expressed as glutathione-S-transferase (GST) fusion proteins in *E. coli*, were incubated with either recombinant DNAJB12 (GST fusion) or ARMC6 (His_6_-tagged), in the presence of [^3^H]-SAM. Proteins were then separated by gel electrophoresis, and methylation detected by fluorography. Hs-, Mm-, Dr-, Tb-, and PiMETTL9 all showed methylation activity on both DNAJB12 and ARMC6, while no MTase activity was observed for CeMETTL9 and DpMETTL9 (Fig. 2*A*), which were also the two enzymes that deviated most from the others in terms of amino acid sequence.

Interestingly, two orthologues, DmMETTL9 and OtMETTL9, displayed distinct substrate preferences. DmMETTL9 methylated ARMC6, while showing no activity on DNAJB12, whereas the opposite was the case for OtMETTL9, which only methylated DNAJB12 (Fig. 2*A*). Noting these interesting differences, we chose to investigate these two enzymes further by generating catalytically inactive mutants as negative controls in subsequent experiments. Mutation of a conserved acidic SAM-binding residue in motif Post I appears to invariably inactivate the 7BS MTases, and the corresponding E174A mutation in HsMETTL9 (Fig. 1*B*), was previously found to abolish MTase activity (7). We introduced the analogous mutations in OtMETTL9 (E145A) and DmMETTL9 (E162A) and indeed observed that the resulting recombinant proteins were devoid of detectable MTase activity (Fig. 2*B*). This confirms that methylation of these substrates is due to specific enzymatic activity of METTL9 orthologues, rather than a contaminating co-purifying *E. coli* MTase.

A recent study reported DNA methylation activity for the CeMETTL9 orthologue (23), prompting us to further investigate whether this enzyme has any protein methylation activity. To this end, we employed HxH-rich recombinant GST fusion fragments of the established HsMETTL9 substrate, zinc transporter ZIP7 (7). These fragments, ZIP7_31-137_ and ZIP7_235-380_, contain 23 and 7 HxH motifs, respectively, facilitating methylation of motifs that are found in neither DNAJB12 nor ARMC6. We also generated a catalytically inactive mutant of CeMETTL9 (E136A) as a negative control. When WT CeMETTL9 was incubated with [^3^H]-SAM and either ZIP7 fragment, we detected robust methylation, while the inactive mutant displayed no activity (Fig. S1*A*). Furthermore, we tested CeMETTL9's ability to methylate single-stranded DNA (ssDNA), as previously reported (23), but found no evidence of DNA methylation (Fig. S1*B*). These findings suggest that, like the other METTL9 orthologues investigated here, CeMETTL9 mainly functions as a protein MTase, while its reported DNA methylation activity may be a marginal moonlighting activity. This is in line with the observation from the same study that MmMETTL9 can also methylate DNA, albeit to a lesser extent than CeMETTL9 (23), despite being a well-established histidine MTase (7), as also demonstrated in Figure 2*A*.

### Dm-, Ot-, and HsMETTL9 display different sequence preferences for peptide methylation

**Figure 3:**
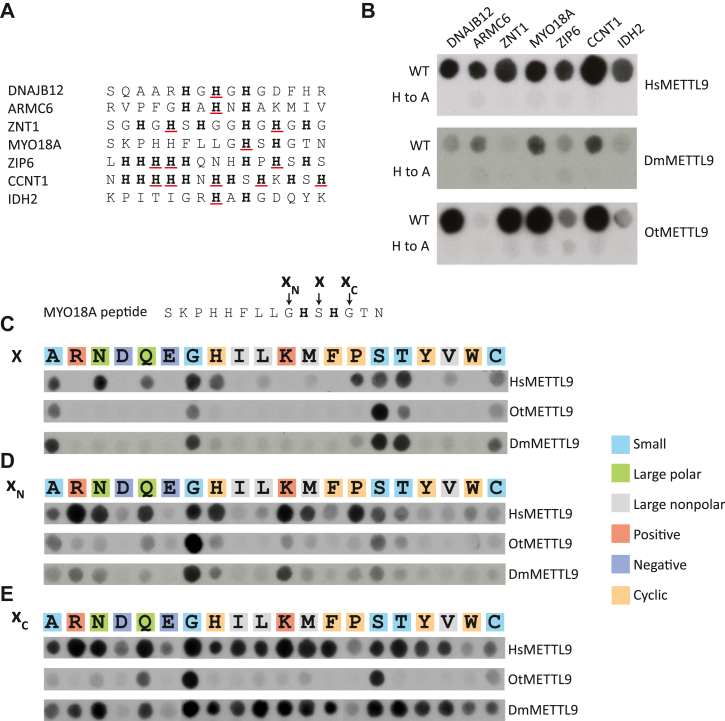
**Peptide array methylation experiments revealing different substrate preferences of the METTL9 orthologues.***A*, Peptide sequence of wild type and H-to-A mutated control peptides investigated in (*B*), H residues mutated to *A* are *underlined*. *B*, activity of HsMETTL9, DmMETTL9 and OtMETTL9 on peptides from seven previously established HsMETTL9 substrates (7). Both WT peptides (*top*) and corresponding peptides with H-to-A mutations (*bottom*) disrupting all HxH motifs were analyzed. C-E, activity of Hs-, Dm- and OtMETTL9 on MYO18A peptides with systematic replacements of the (C) middle (X) residue (S in the WT sequence), (D) the N-terminally flanking (X_N_) residue (G in the WT sequence), and *(E)* the C-terminally flanking (Xc) residue (G in the WT sequence). Note that the original images, which can be found in Fig. S2, have been joined between E and G, and F and P to display the data as continuous rows.

We have previously observed that HsMETTL9 is active on short HxH-containing peptides, and in this work set out to use such peptide substrates to further investigate the sequence specificity of DmMETTL9 and OtMETTL9. To this end, we employed peptide SPOT arrays with immobilized 15-mer peptides, first investigating seven human HxH-containing sequences previously found to be HsMETTL9 substrates (7). As negative controls, we included corresponding H-to-A mutated peptides lacking any HxH motifs (Fig. 3*A*). As expected, HsMETTL9 methylated all seven peptides, albeit with different efficiency, whereas both DmMETTL9 and OtMETTL9 only methylated a subset of the peptides (Fig. 3*B*). In agreement with the results obtained with full-length proteins (Fig. 2), OtMETTL9 showed negligible activity on the ARMC6-derived peptide, whereas the activity of DmMETTL9 was reduced on the DNAJB12 peptide. Moreover, DmMETTL9 exhibited low activity on a ZNT1 peptide, which was an excellent substrate for both Ot- and HsMETTL9. In addition, both Dm- and OtMETTL9 showed relatively low activity on peptides derived from IDH2 and ZIP6 (Fig. 3*B*). Thus, the results of the above experiments further confirm that the substrate specificities of the three investigated METTL9 proteins are different.

### Dm- and OtMETTL9 tolerate fewer amino acid substitutions in and flanking the HxH motif than HsMETTL9

Given the observed differences in substrate specificity between the METTL9 orthologues, we wanted to investigate how the identity of the middle “X” residue of the HxH METTL9 recognition motifs affects methylation. For this, we chose the myosin-XVIIIa (MYO18A) peptide as substrate, because it is an excellent substrate of all three (Hs, Ot, and Dm) enzymes, and only contains a single HxH motif (Fig. 3, *A* and *B*). Previously, we (7) and others (8) have observed that HsMETTL9 prefers small residues (A, N, G, S, T, C) in the middle position of the HxH motif in different peptides, and our present data are in good agreement with this. In addition, the residues Q, H, and P were also well tolerated at this position in the context of the MYO18A peptide, consistent with previous observations (7) (Fig. 3*C*). The Ot- and DmMETTL9 enzymes, however, displayed more stringent sequence requirements, exclusively methylating sequences containing A, G, S, T, or C, but not the bulkier N, Q, H, or P as the middle residue (Fig. 3*C*).

When we investigated the effects of replacing the residues flanking the HxH motif (X_N_: N-terminally flanking; X_C_: C-terminally flanking) in the MYO18A peptide, we again observed clear differences in the sequence preferences of the orthologues. For the X_N_ position (G in the WT sequence), HsMETTL9 methylated many of the modified peptides but, similarly to our previous findings (7), it was less active on peptides containing the negatively charged D and E residues, the large nonpolar residues I, L, and V, as well as the aromatic Y, F, and W (Fig. 3*D*). Surprisingly, in the MYO18A peptide, P was one of the preferred X_N_ residues for HsMETTL9, a preference not observed in previous studies (7, 8). This variability underlines our previous finding that the activity of HsMETTL9 is also affected by sequence features other than the HxH motif and its directly flanking residues. As for the orthologues, both Dm- and OtMETTL9 displayed a much more stringent requirement for the X_N_ position, showing a strong preference for G, the original residue in the WT sequence, over any other residue, with DmMETTL9 additionally efficiently methylating the K-replaced peptide (Fig. 3*D*).

At the X_C_ position (G in the WT sequence), HsMETTL9 tolerated almost any replacement besides P, W, C, and the negative D and E, all of which greatly reduced its activity (Fig. 3*E*), again in line with our previous findings (7). Strikingly, DmMETTL9 displayed a methylation pattern almost identical to that of HsMETTL9. OtMETTL9, however, was very sensitive to the identity of the residue at this position, only methylating peptides containing G, S, or, to a lesser extent, Q (Fig. 3*E*).

In summary, compared to HsMETTL9, Dm- and Ot-METTL9 displayed more stringent requirements for the identity of the X- and X_N_-residues of the MYO18A substrate peptide, and, in addition, OtMETTL9 was especially sensitive to replacement of the Gly residue at the X_C_ position.

### OtMETTL9 generates 1MH on DNAJB12

**Figure 4:**
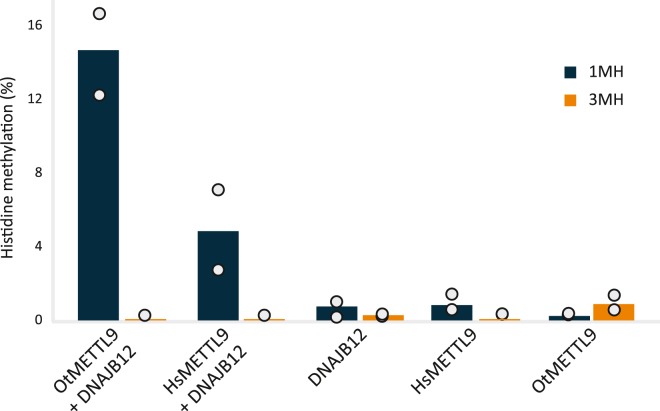
**OtMETTL9 generates 1MH.** After methylation of GST-DNAJB12 with either GST-Ot- or GST-HsMETTL9 in the presence of SAM, reaction products were hydrolyzed into single amino acids, which were analyzed by mass spectrometry. The amounts of 1MH and 3MH are expressed as percentages of total histidine. Reactions containing only substrate (DNAJB12) or enzyme (Hs- or OtMETTL9) represent negative controls. Data are represented as the mean with individual values of duplicates.

Since HsMETTL9 generates 1MH, we set out to investigate whether this is also the case for OtMETTL9. For this experiment, recombinant DNAJB12 was incubated with OtMETTL9 or HsMETTL9 in the presence of non-labeled SAM. Corresponding control reactions containing either the substrate or the MTase alone were included. The samples were then hydrolyzed into amino acids and analyzed by mass spectrometry (MS) for the presence of methylhistidine. While the controls showed only background amounts of methylhistidine, DNAJB12 incubated with either Hs- or OtMETTL9 displayed a clear increase in 1MH, indicating that OtMETTL9, like HsMETTL9, generates 1MH (Fig. 4).

### Structural analysis of OtMETTL9

**Figure 5:**
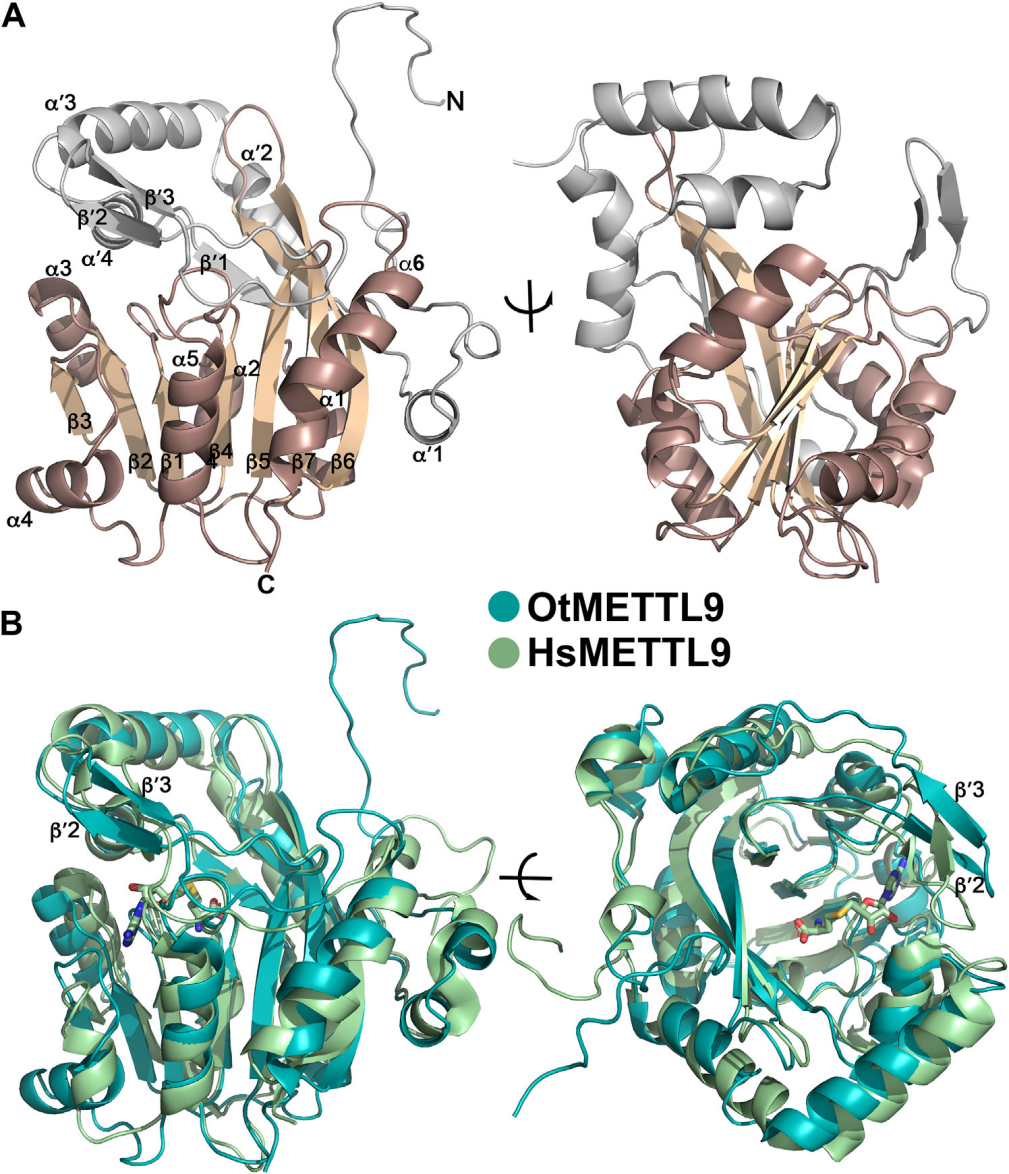
**Comparison of the structures of OtMETTL9 and HsMETTL9.***A*, the crystal structure of OtMETTL9 shown in two orientations with the 7BS part colored in *brown* (α-helices) and *wheat* (β-strands) with other structural elements in *gray*. *B*, comparison of OtMETTL9 and HsMETTL9 (PDB 7YF2 with SAH) structures in two orientations, demonstrating the different positioning of strands β′2 and β′3. OtMETTL9 in *teal*, HsMETTL9 in *pale green*.

To investigate whether any structural differences could explain the distinct substrate specificity of OtMETTL9, we solved its apo crystal structure and compared it to the recently published structures of HsMETTL9 (16, 17) (Fig. 5). Like HsMETTL9, the OtMETTL9 structure is a monomer, adopting the canonical 7BS MTase fold consisting of seven β-strands (β1-β7) that form a twisted sheet of characteristic topology, alternating with six α helices (α1-α6) located on both sides of the β-sheet (Fig. 5*A*). The overall structure is very similar to HsMETTL9 with a root-mean square deviation (RMSD) of only 2.1 Å (Fig. 5*B*). Like HsMETTL9, OtMETTL9 contains additional structural elements (β′1-β′3, α′1-α′4) outside of the core 7BS fold, which are typically involved in substrate recognition (22). Among them, the region containing β′2-β′3 has a slightly different orientation in OtMETTL9 than in HsMETTL9 as will be discussed later (Fig. 5*B*). It also contains the β7 and β6 strands, which are particularly long in METTL9 enzymes as compared to other 7BS MTases, and the elongated part of β6 forms hydrogen bonds with the short β-strand, β′1, thereby extending the canonical 7BS fold (Fig. 5).

**Figure 6:**
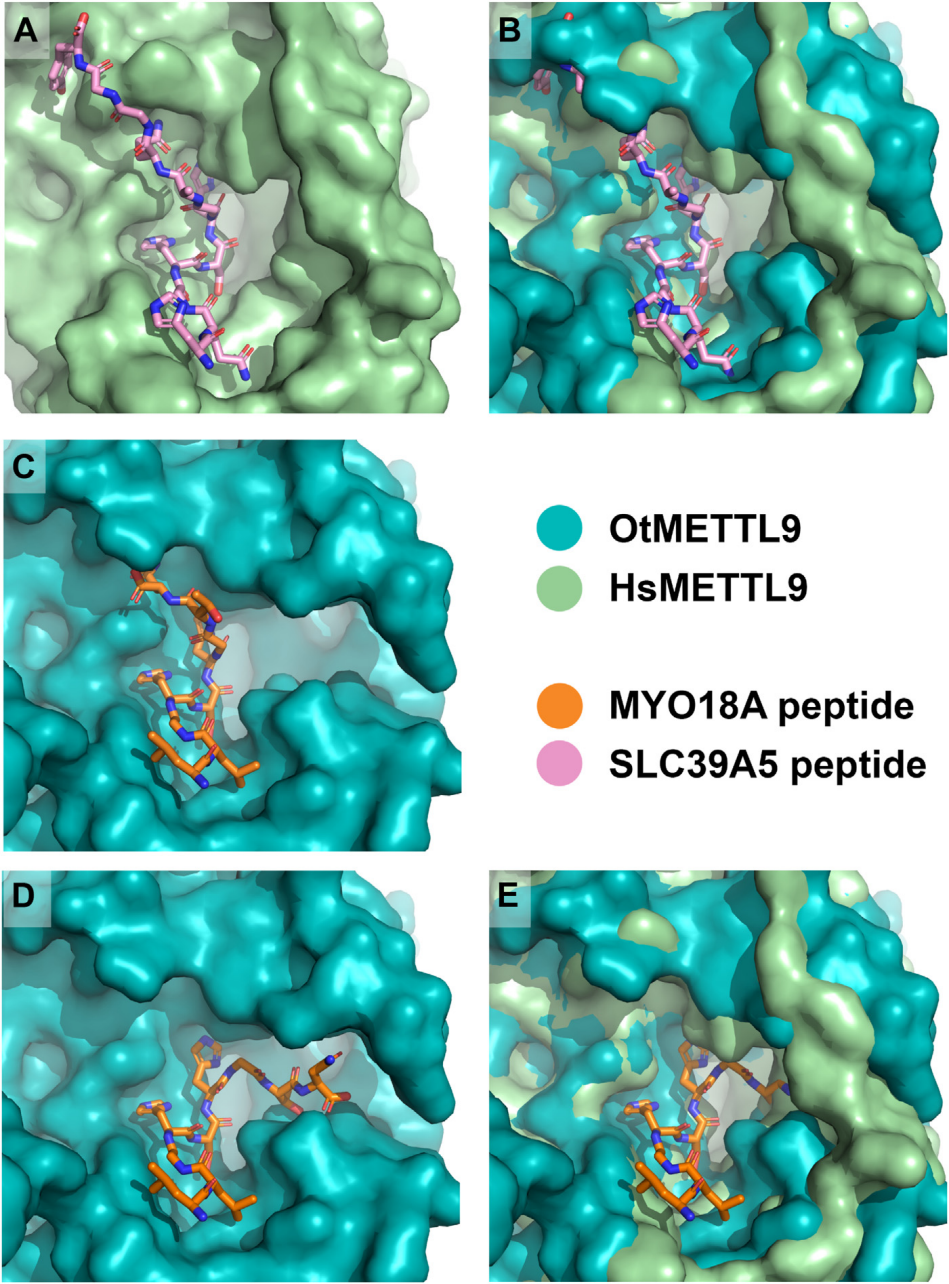
**Putative OtMETTL9 substrate binding and interactions**. Surface representation of METTL9 structures binding peptides. A and B, binding of the SLC39A5 (ZIP5) peptide in the (A) straight groove of the HsMETTL9 structure (PDB 7YF2), and (B) overlaid with OtMETTL9 surface representation, showing OtMETTL9 steric clashes with the C-terminal part of the peptide. C and D, OtMETTL9 with the MYO18A peptide modelled in the (C) PDB 7YF2-like orientation (straight binding groove), and (D) in the alternative orientation using the side pocket of OtMETTL9 (C-shaped binding groove). *E*, overlay of HsMETTL9 and OtMETTL9 surfaces with the MYO18A peptide in the C-shaped binding orientation, showing that this groove is blocked in HsMETTL9. Peptides are represented as *pink* (SLC39A5) or *orange* (MYO18A) sticks, OtMETTL9 surface in *teal*, HsMETTL9 in *pale green*.

When we attempted to dock the SLC39A5 (ZIP5) peptide previously co-crystallized with HsMETTL9 (PDB 7YF2, (16)) into our OtMETTL9 structure, we noticed that this peptide could not be accommodated in the exact same groove as in HsMETTL9, with steric hindrances from the main chains of S250 and A251 preventing proper positioning of the residues on the C-terminal side of the peptide (Fig. 6, *A* and *B*). We thus modeled a shorter version of the MYO18A peptide used in the arrays, LLGHSHGTN, into the active site of OtMETTL9 by docking the SLC39A5 peptide of corresponding length from HsMETTL9 with FlexPepDock, mutating it to the appropriate sequence and performing the built-in optimization (24) (Fig. 6*C*). We also noticed that the binding surface of OtMETTL9 could instead extend into an adjacent side pocket, where peptide residues located C-terminally of the HxH-motif could bind, creating a bent, C-shaped, binding groove, as opposed to the straight groove of HsMETTL9 (Fig. 6*D*, and Fig. S3). For HsMETTL9, access to this side pocket is blocked by strands β′2 and β′3, which are positioned differently from OtMETTL9 (Figs. 5B and 6*E*). Binding of the peptide in the C-shaped groove in OtMETTL9 may impose additional constraints on the identity of the X_c_ residue, which may explain the more stringent requirement (small residues G or S) for peptide methylation activity of the OtMETTL9 enzyme as compared to HsMETTL9 (Fig. 3*E*).

A limitation of our study is that, in our apo OtMETTL9 structure, we cannot rule out potential conformational changes in the binding pocket upon peptide binding. For the published HsMETTL9 structures, however, peptide binding did not lead to large changes in the configuration of the binding groove. Notably, the side pocket present in OtMETTL9 remained blocked in both the apo- and the peptide-bound HsMETTL9 structures (Fig. 6*C*, and PDB 8GZF apo vs PDB 8GZE with SLC39A7 (ZIP7) peptide (17)).

**Figure 7:**
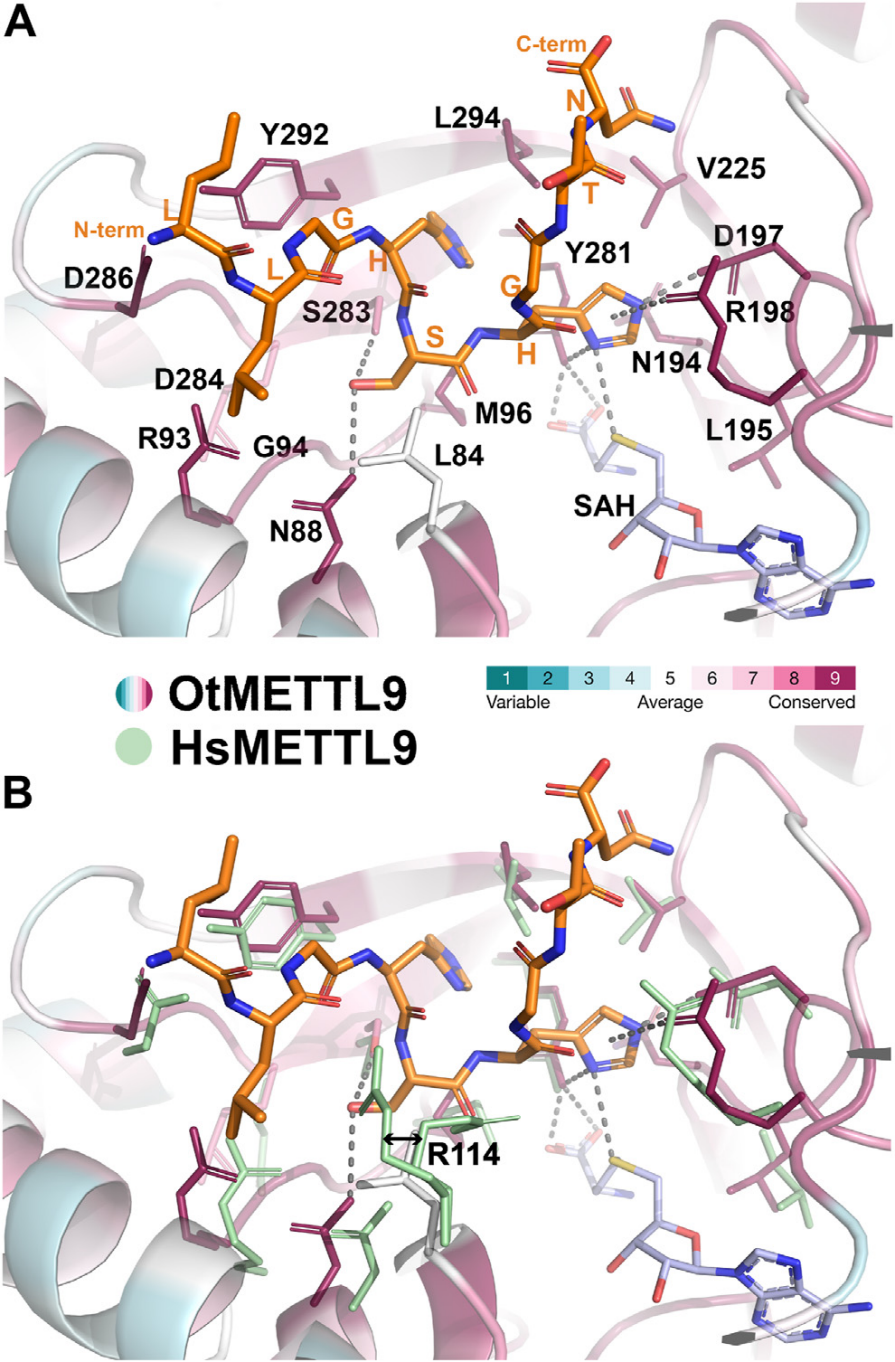
**Peptide-interacting residues in OtMETTL9 and HsMETTL9.** A and B, MYO18A peptide modelled into the OtMETTL9 structure and HsMETTL9 structure, (A) showing interacting residues for OtMETTL9 only, and (B) overlayed with HsMETTL9 binding pocket residues. The MYO18A peptide was mutated from the SLC39A5 (ZIP5) peptide (PDB 7YF2) and optimized with FlexPepDock. The comparison shows high conservation of the identity and orientation of the residues interacting with the peptide. The figure also shows the movement (arrow) of R114 in the apo (PDB 8GZF) vs peptide-bound (PDB 8GZE) HsMETTL9 structure, this residue is modelled as A in PDB 7YF2, and is not conserved in OtMETTL9 (L84). OtMETTL9 is colored by conservation with ConSurf, HsMETTL9 residues in teal, the peptide in orange.

We also looked at specific residues in OtMETTL9 that interact with the X and X_N_ positions in the modelled MYO18A peptide and compared them with those in HsMETTL9 (16, 17) (Fig. 7). Most of the residues that are in contact with the X or X_N_ positions in the HsMETTL9 structures (16, 17) are identical and have a similar orientation in the OtMETTL9 structure, such as N88 (Hs N118), R93 (Hs R123), G94 (Hs G124), M96 (Hs M126), R198 (Hs R214), D286 (Hs D300), and Y292 (Hs Y306), whereas S283 has similar properties to the human C297. The only difference is OtMETTL9 L84, corresponding to the human R114, which moves upon peptide binding (17) and interacts with both X and X_N_ (Fig. 7*B*). Interestingly, this position is not conserved in several other METTL9 homologues that we have investigated, including DmMETTL9 (Q in Dm, Fig. 1*B*).

The HsMETTL9 residues interacting with X_C_ were examined in (17), where, of note, the peptide residue at the X_C_ position, E (E69 in ZIP7), is not among the preferred ones for HsMETTL9 ((7) and this study) so additional adjustments may have been required by the enzyme to accommodate it. Indeed, two HsMETTL9 residues, H235 and W255, were shown to flip inward to promote E69 interaction (17). Interestingly, these residues, corresponding to R229 and R239 in OtMETTL9, are not conserved. In addition, when binding in the C-shaped groove of OtMETTL9, the X_C_ residue of the MYO18A peptide would have a different set of interactions than in the HsMETTL9-like straight groove (Fig. S3).

In conclusion, there are only a few differences in the specific residues previously shown for HsMETTL9 to interact with X and X_N_ positions of substrate peptides, as compared to OtMETTL9. The main structural difference lies in the shape of the binding pocket, which may restrict the accommodation of the peptide residues located C-terminally to the HxH motif.

## Conclusion

HsMETTL9 accounts for roughly half of the 1MH content in the proteome and methylates histidines within HxH motifs, with its activity further influenced by the identity of the middle X residue as well as the flanking residues (X_N_ and X_C_). In this study, we have demonstrated that Ot- and DmMETTL9 are functional METTL9 orthologues with distinct substrate specificities most likely adapted to the biologically most relevant substrates in the corresponding species. Using peptide array replacement analysis on a previously unexamined peptide substrate derived from the MYO18A protein, we systematically compared the sequence requirements for methylation by HsMETTL9, OtMETTL9, and DmMETTL9. Our findings reveal that, compared to HsMETTL9, Ot- and DmMETTL9 have a more stringent requirement for smaller residues at the X_N_ and X positions of the substrate peptide, and OtMETTL9 is highly sensitive to the identity of the X_C_ residue. We also solved the apo structure of OtMETTL9 and, through peptide modeling, identified several differences with peptide-bound HsMETTL9. In particular, the potential presence of a C-shaped binding groove in OtMETTL9 may place additional restrictions on the peptide residues following the HxH motif and contribute to the stringent OtMETTL9 X_C_-specificity observed in the peptide array.

## Experimental procedures

### Sequence alignments

Multiple protein sequence alignments were performed using the Muscle algorithm embedded in Jalview (25).

### Cloning and mutagenesis

The pGEX-6P-2- and pET28a(+)-derived plasmids encoding GST-DNAJB12 (1–243 aa), GST-ZIP7 (31–137 aa and 235–380 aa), His_6_-ARMC6, and His_6_-HsMETTL9 were generated in a previous study (7). Constructs of pGEX-6P-2 encoding GST-METTL9 orthologues and mutants, as well as pET-22b(+) encoding untagged OtMETTL9, were codon optimized for bacterial expression and custom ordered from GenScript. All generated constructs were sequence-verified.

### Expression and purification of recombinant proteins

Protein expression plasmids were transformed into competent *E. coli* BL21-CodonPlus (DE3)-RIPL cells (Agilent Technologies) or, in the case of pET-22b(+) encoding untagged OtMETTL9, into competent *E. coli* One ShotTM BL21 (DE3) cells (Invitrogen, Thermo Fischer Scientific). After overnight growth in LB medium, the cultures were inoculated into a larger volume of Terrific Broth medium containing the appropriate antibiotics. Protein expression was induced by adding isopropyl-β-D-thiogalactopyranoside (IPTG) (Thermo Fischer Scientific) to a final concentration of 0.1 to 0.5 mM at OD600 = 0.8, and the cultures were incubated for 12 to 16 h at 18 °C with vigorous shaking before cells were harvested and stored at −20 °C. For GST-tagged proteins, bacteria were lysed in 50 mM Tris-HCl pH 7.6, 500 mM NaCl, and 0.5% Triton X-100 supplemented with 1 mg/ml lysozyme, 1 mM DTT, and 1x cOmplete Protease Inhibitor Cocktail (Roche), and sonicated. Proteins were loaded on a Poly-Prep Chromatography Column (BioRad) and eluted with 10 mM reduced glutathione, 50 mM Tris pH 8.9, and 500 mM NaCl. For His_6_-tagged proteins, bacteria were lysed in 50 mM Tris-HCl pH 8.0, 500 mM NaCl, 5% (v/v) glycerol, 1% (v/v) Triton X-100, 5 mM 2-mercaptoethonol, 1 mg/ml lysozyme, 10 mM imidazole, and 1x cOmplete (EDTA-free) Protease Inhibitor Cocktail (Roche), and sonicated. Purification was performed using Ni-NTA-agarose (Qiagen) and Poly-Prep Chromatography Columns (BioRad). Proteins were eluted using 50 mM Tris pH 8.0, 500 mM NaCl, 5% glycerol, 5 mM β-mercaptoethanol, and 300 mM imidazole. Eluted proteins from Glutathione Sepharose 4B and Ni-NTA-agarose purification were buffer exchanged to 50 mM Tris-HCl pH 8.0, 300 mM NaCl, 5% glycerol, and 1 mM DTT using centrifugal ultrafiltration units (10 kDa MWCO) (Sartorius).

For crystallization, the GST tag of GST-OtMETTL9 was cleaved with PreScission Protease (Merck), which leaves a GPLGS sequence at the N terminus of the protein. In short, for each 1 mg protein, 20 U PreScission protease, 20 μl pre-washed resin, and 2 ml Cleavage Buffer (50 mM Tris pH 7.6, 500 mM NaCl, 1 mM DTT, 5 mM EDTA) were incubated for 24 h at 4 ^°^C. Samples were loaded on Poly-Prep Chromatography Columns (BioRad), and the flow-through was collected. After cleavage of the GST tag, OtMETTL9 was buffer exchanged to 50 mM Tris-HCl pH 7.5, 1 mM DTT using centrifugal ultrafiltration units (10 kDa MWCO) (Sartorius), applied on a HiTrap Q HP anion exchange column (Cytiva), and eluted with a linear 0 to 0.2 M KCl gradient. For non-tagged OtMETTL9, bacterial cells were thawed and resuspended in 50 mM Tris-HCl pH 7.5, 150 mM NaCl, 2 mM DTT, 5 μg/ml DNase, and 1x cOmplete Protease Inhibitor Cocktail (Roche) in a 1:4 cell wet weight to buffer ratio and lysed by sonication. Protein was precipitated with ammonium sulfate ((NH_4_)_2_SO_4_) to a final concentration of 0.45 g/ml, dissolved in 50 mM Tris-HCl pH 7.5, 1 mM DTT, and desalted through dialysis (SnakeSkin dialysis tubing, 10 kDa MWCO, ThermoFisher Scientific) in the same buffer. Desalted protein was applied to a HiTrap Q HP anion exchange column (Cytiva) and eluted with a linear 0 to 0.2 M KCl gradient. As a final polishing step, protein was purified on a Superdex 200 Increase 10/300 Gl (Cytiva) in 50 mM Hepes pH 7.5, 100 mM NaCl. The concentration of OtMETTL9 was estimated using the extinction coefficient ε280 = 22.46 mM^−1^ cm^−1^. All proteins were stored at −80 °C.

### *In vitro* MTase assays

For amino acid analysis and fluorographic visualization of methylated proteins, the reaction buffer was (50 mM Tris-HCl pH 7.5, 50 mM NaCl, 5 mM EDTA), and the methyl donor SAM was either unlabeled (1 mM) or radiolabeled [0.64 μM ^3^H-SAM (Perkin–Elmer, specific activity = ∼77–78 Ci/mmol)], respectively.

For each reaction, the enzyme concentration was ∼1 μM. For protein substrates, 10 μg was used, and for the cell fractions and whole cell lysate, 20 μg total protein was used per reaction. The total reaction volume was 10 μl or 20 μl. Incubation was performed for 1h at 37 °C with light shaking. For fluorographic analysis, the reaction was stopped with NuPAGE loading buffer (Thermo Fisher), and the products were separated by SDS-PAGE (Thermo Fisher) before transfer to a PVDF membrane (Merck). The membrane was stained with Ponceau S for visualization of proteins and sprayed with an enhancer scintillation spray (57.5% 2-methylnaphthalene, 40% pentylacetate, 2.5% diphenyloxazole) to visualize the radioactive signal. The membrane was exposed to an Eastman Kodak Co. Bio-Max MS film (Sigma-Aldrich) at −80 °C.

For *in vitro* DNA MTase activity assays, 2 μM biotin-labelled oligonucleotides in reaction buffer (50 mM Tris pH 7.5, 80 mM KCl, 2.5 mM MgCl_2_, 0.2 mM DTT, 0.64 μM ^3^H-SAM) were incubated with 1 μM WT or Mut GST-CeMETTL9, in a total volume of 50 μl, for 2 h at 20 °C. Samples with no added enzyme were used as negative control. The sequences of biotinylated oligonucleotides were acquired from (23) (ssDNA WT: CGTGCTTGCTACTGGTGGGG**A**GAATGCATGCTACTGGTGC-Biotin and ssDNA mut: CGTGCTTGCTACTGGTGGGG**T**GAATGCATGCTACTGGTGC-Biotin). Following incubation, the volume was adjusted to 200 μl with H_2_O and incubated with 10 μl prewashed Dynabeads MyOne Streptavidin C1 beads for 1 h at 4 °C. Beads were then washed four times with TE buffer and incorporated radioactivity was quantified using liquid scintillation counting using Ultima Gold XR (PerkinElmer).

### Amino acid analysis

*In vitro* methylation assay with unlabeled SAM was performed with GST-OtMETTL9, GST-HsMETTL9, and GST-DNAJB12, or the enzymes and substrate alone, as described above. Reaction products were transferred to vacuum hydrolysis tubes (Thermo Fisher Scientific), and 200 μl Sequencing Grade 6 M HCl (Thermo Fisher Scientific) was added. Hydrolysis was performed under vacuum at 110 °C for ∼48 h. The acid was evaporated at 50 °C, and the pellet resuspended in 500 μl H_2_O, filtered through a 0.22-μm syringe filter (Millex-GP), and dehydrated at 50 °C.

Dried samples were resuspended in 40 μl 0.1% formic acid, and 1MH and 3MH were quantified using an LC-MS/MS method. Analytes were separated using an Agilent Infinity II UHPLC system with a Primesep200 column (150 × 2.1 mm ID). The analytes were separated using a flow rate of 0.23 ml/min and a gradient from 0 to 80% acetonitrile, 0.1% formic acid over 10 min, and detected using an Agilent 6495 Triple quadrupole system operating in positive electrospray mode. The following analytes were detected using the indicated mass transitions: 1MH (170.1–96.0), 3MH (170.1–123.9), His (156.1–83.0), and Arg (175.1–70.0). Calibration curves used for quantification were established from 1 nM to 500 nM for all analytes.

### Protein crystallization

All crystallization screening of OtMETTL9 was performed with a Mosquito crystallization robot (SPT Labtech) at 4 °C. For crystallization, both the GST-cleaved and non-tagged OtMETTL9 were used. Crystals of non-tagged OtMETTL9 were obtained with condition H3 from the Morpheus crystallization screen (Molecular Dimensions) (0.1 M imidazole/MES monohydrate pH 6.5, 0.02 M DL-glutamic acid monohydrate, 0.02 M DL-alanine, 0.02 M glycine, 0.02 M DL-lysine monohydrochloride, 0.02 M DL-serine, 20% v/v glycerol, 10% w/v polyethylene glycol (PEG) 4000) and (27 mg/ml non-tagged OtMETTL9) and with condition F1 from the Index crystallization screen (Hampton Research) (0.1 M Hepes pH 7.5, 0.2 M L-proline, 10% w/v PEG 3350) and (38 mg/ml GST-cleaved OtMETTL9). All crystals were grown at 4 °C and flash-frozen in liquid N_2_ prior to data collection.

### Crystal data collection, processing, and refinement

Diffraction data for both crystallization conditions were collected at beamline ID30B at the European Synchrotron Radiation Facility (ESRF), Grenoble, France. The raw data (diffraction data) were archived in the ESRF depository (https://doi.org/10.15151/ESRF-ES-874802477). Crystals obtained from both crystallization conditions diffracted to approximately 3 Å, but the highest resolution dataset (Index crystallization screen) was chosen for further analysis. Diffraction data were indexed and integrated through auto-processing with autoPROC (26) and XDS (27) and scaled and merged with Aimless in the CCP4 package (28). The structure was solved by molecular replacement with PHASER (29) using the AlphaFold model of OtMETTL9 (Q01C52-F1-model_v3) from the AlphaFold Protein Structure Database as a search model removing the first 10 N-terminal residues due to its predicted loop structure (30, 31). Initial refinement was performed using restrained refinement in REFMAC5 (32) followed by several cycles of refinement with phenix.refine (33) in the Phenix (34) suite and model building in Coot (34). Model validation was performed using MolProbity (35). The absorbed X-ray dose was calculated with the program RADDOSE-3D (36). The final data collection and refinement statistics are listed in Table S1. All structure figures were prepared using PyMOL (37), and the secondary structure assignment for OtMETTL9 was performed by the program DSSP, NKI version 4.0 (38).

### Analysis of structure similarities and conserved residues

Comparison with similar structures of the OtMETTL9 in the Protein Data Bank (PDB) and root-mean-square deviations (RMSD) calculation was performed using the DALI (Distance-matrix Alignment) protein structure comparison server using the OtMETTL9 structure as a search template (39). To evaluate the degree of conservation of residues in the OtMETTL9 structure, ConSurf (40, 41, 42, 43) was run on the OtMETTL9 structure. The run was based on the homologue search algorithm HMMER, searching sequences from UniRef90, and multiple sequence alignment with MAFFT. This gave 324 homologous sequences passing the standard threshold, and ConSurf used a sample of 150 sequences that represented the list of homologous sequences to map the conservation on a 9-bin scale from turquoise (most variable) to maroon (most conserved). The conservation color coding was then mapped onto the OtMETTL9 crystal structure, and figures were generated with PyMOL.

### Protein-ligand docking

To explore the active site of OtMETTL9 and potential interaction with the MYO18A peptide, the SLC39A5 peptide (HQGHSHGHQGGY) from a co-structure with HsMETTL9 (PDB 7YF2) was used as a starting point by aligning Ot- and Hs-METTL9 structures. The full SLC39A5 peptide from [7YF2] did not fit into the OtMETTL9 groove due to the position of S250 and A251 in OtMETTL9. Therefore, the GGY end of the peptide was removed, and the remaining residues were mutated to those of MYO18A in Coot with an acceptable rotamer orientation. The resulting peptide (LLGHSHGTN) was then optimized using the FlexPepDock minimization mode in Rosetta (24). To obtain the alternative peptide binding in the C-shaped groove, the peptide was manually adjusted and then optimized similarly with FlexPepDock. For schematic visualization of protein–ligand interactions between the OtMETTL9 structure and the docked MYO18A peptides, the program LigPlot^+^ was used with default parameters to show hydrogen bonds, ionic interactions, and hydrophobic contacts represented by dashed lines and arcs with spokes (44).

### Peptide array synthesis and methylation

The peptide arrays were synthesized on a cellulose membrane using the SPOT synthesis method (45, 46, 47) with an Autospot peptide array synthesizer (Intavis AG, Köln, Germany). After synthesis, the membranes were pre-incubated in methylation buffer (50 mM Tris/HCl pH 8, 300 mM NaCl, 5% glycerol and 1 mM DTT) for 5 min. Thereafter, the arrays were incubated for 60 min at room temperature in methylation buffer containing 100 nM HsMETTL9, 100 nM OtMETTL9 or 600 nM DmMETTL9 and [^3^H]-labelled SAM (PerkinElmer Inc.) (0.5 mCi/ml; 82.3 Ci/mmol specific activity). After methylation, the membranes were washed 5 times for 5 min in wash buffer (100 mM NH_4_HCO_3_, 1% SDS) and incubated in Amplify NAMP100 V (GE Healthcare) for 5 min. This was followed by the exposure of the membranes to a Hyperfilm high performance autoradiography (GE Healthcare) film at −80 °C in the dark. The films were developed with an Optimax Typ TR machine after different exposure times. Exposure times for X replacements: 2 days (Hs), 2 weeks (Ot), 5 weeks (Dm); X_N_-replacements: 1 week (Hs), 1 week (Ot), 5 weeks (Dm); X_C_-replacements: 1 week (Hs), 2 weeks (Ot), and 5 weeks (Dm).

## Data availability

The structure of OtMETTL9 has been deposited in the Protein Data Bank (PDB 8BVI).

## Supporting information

This article contains supporting information (23).

## Conflict of interest

The authors declare that they have no conflicts of interest with the contents of this article.
