# Supplementary material for: Orthologues of the human protein histidine methyltransferase METTL9 display distinct substrate specificities
Source: J Biol Chem. 2025 May 30;301(7):110318. doi: 10.1016/j.jbc.2025.110318 (PMC12269839; doi:10.1016/j.jbc.2025.110318)
Supplement: Supporting Information [file mmc1.docx]

**Supporting Information**

Orthologues of the human protein histidine methyltransferase METTL9 display distinct substrate specificities

Lisa Schroer^1^, Sara Weirich^2^, Marta Hammerstad^1^, Hans-Petter Hersleth^1^, Ida Andrietta Grønsberg^1^, Lars Hagen^3,4,5^, Geir Slupphaug^3,4,5^, Jedrzej Mieczyslaw Malecki^1,6^, Albert Jeltsch^2*^, Pål Ø. Falnes^1,6**^, Erna Davydova^1,6***^

Figure S1

**A**


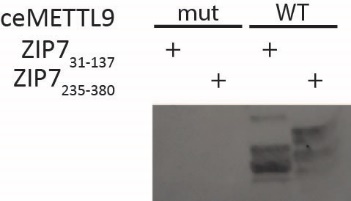


**B**


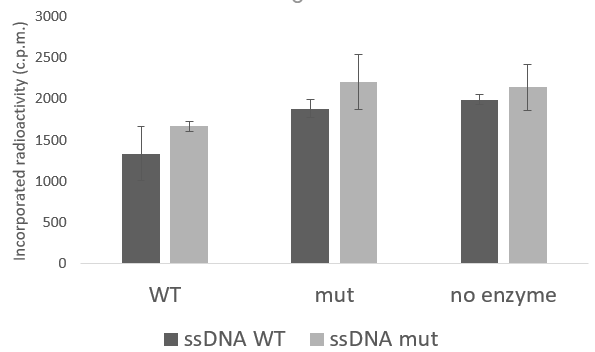


**Figure S1:** **ceMETTL9 is a protein methyltransferase. (A)** In vitro methylation activity of WT or the E136A mutant (mut) of CeMETTL9 on GST-tagged fragments of the zinc transporter protein ZIP7: ZIP7_31-137_ and ZIP7_235-380_. **(B)** DNA methylation activity of WT or mut CeMETTL9 on either the WT or mutant ssDNA substrate taken from [23].

ssDNA WT: CGTGCTTGCTACTGGTGGGG**A**GAATGCATGCTACTGGTGC-Biotin

ssDNA mut: CGTGCTTGCTACTGGTGGGG**T**GAATGCATGCTACTGGTGC-Biotin.

Figure S2


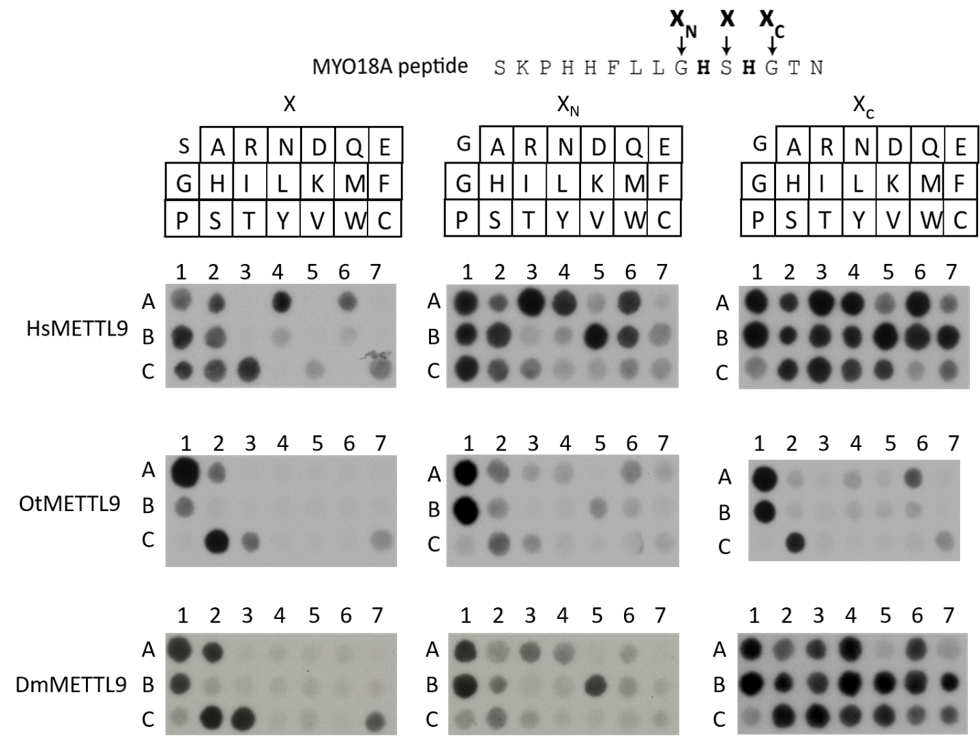


**Figure S2.** **Original images of peptide arrays from Figure 3C, 3D and 3E.** Activity of Hs-, Dm- and Ot-METTL9 on MYO18A peptides where the middle (X, left panel), N-terminally flanking (X_N_, middle panel), or C-terminally flanking (X_C_, right panel) residue was systematically replaced by the other amino acids according to the charts at the top of the figure. The wild type peptide sequence is shown at the top.

Figure S3

**
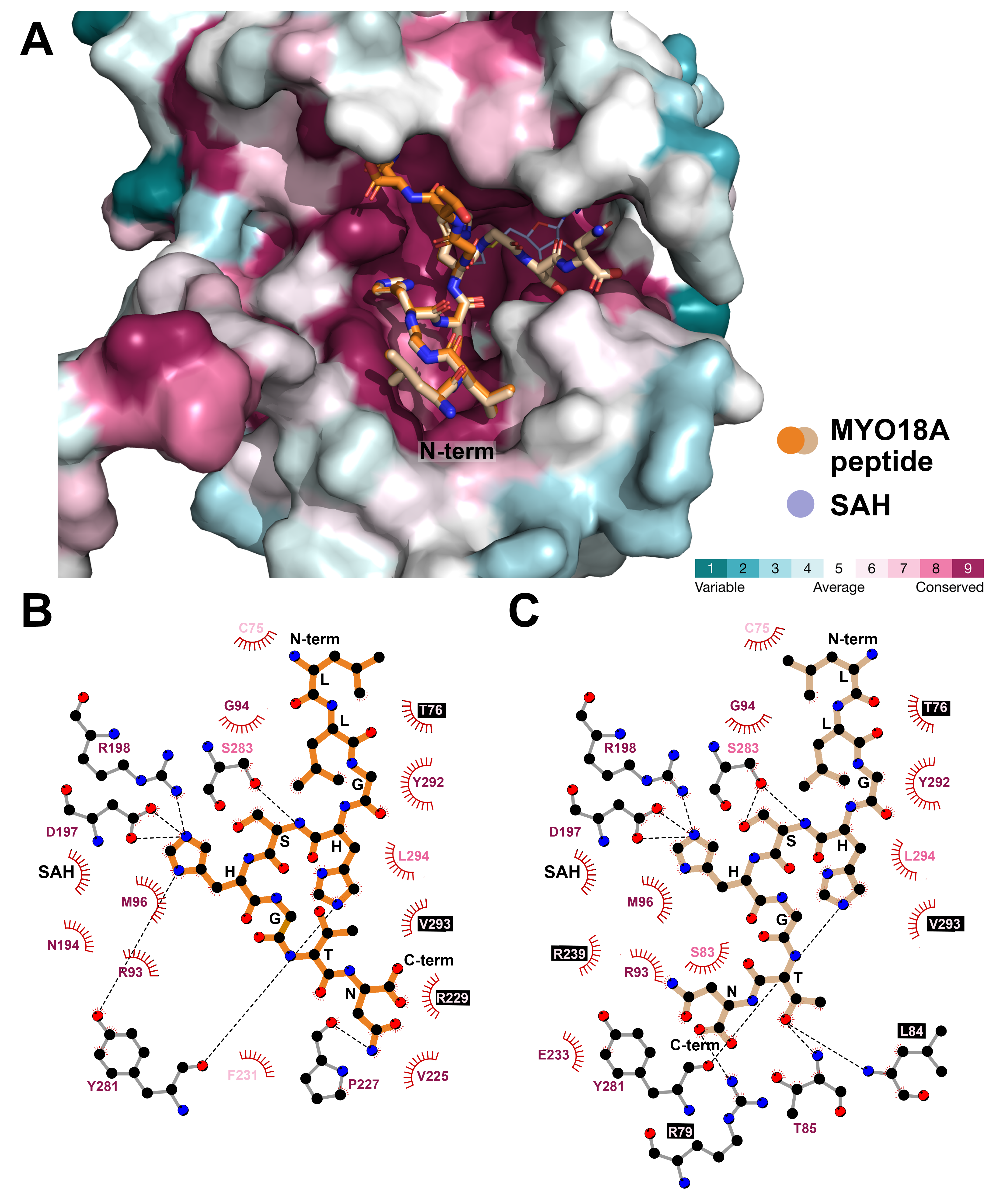
**

**Figure S3. Predicted interactions of the MYO18A peptide in different pockets of OtMETTL9. (A)** Surface view of OtMETTL9 with the MYO18A peptide docked in different conformations. LigPlot^+^ images showing the ConSurf-colored conserved residues lining the putative OtMETTL9 substrate binding pocket with the MYO18A peptide docked either in the HsMETTL9-like conformation **(B)** or in the alternative “side pocket” **(C)**. Hydrogen bonds, ionic interactions, and hydrophobic interactions represented by dashed lines and arcs with spokes.

Table S1. Crystal Data Collection and Refinement Statistics.

|  | OtMETTL9 |
| --- | --- |
| Data collection |  |
| X-ray source | ID30B ESRF |
| Detector | Eiger2 X 9M |
| Wavelength (Å) | 0.918402 |
| Space group | P2_1_3 |
| *a*, *b*, *c* (Å) | 106.9, 106.9, 106.9 |
| α, β, γ (°) | 90, 90, 90 |
| Type | Standard rotation |
| Rotation range per image (°) | 0.1 |
| Total rotation range (°) | 90 |
| Exposure time per image (s) | 0.03 |
| Flux (ph/s) / Transmission (%) | 1.3⋅10^12^ / 6.9 |
| Beam size (µm^2^) | 30 × 30 |
| Crystal size (µm^3^) | 60 × 60 × 80 |
| Average diffraction weighted X-ray dose (MGy) | 4.9 |
| Mosaicity (°) | 0.06 |
| Resolution range (Å) | 47.8-3.10 (3.31-3.10) |
| Total no. of reflections | 72015 |
| No. of unique reflections | 7642 |
| *R*_meas_ | 0.124 (2.010) |
| *R*_merge_ | 0.117 (1.900) |
| R_pim_ | 0.041 (0.648) |
| Completeness (%) | 100.0 (100.0) |
| Multiplicity | 9.4 (9.4) |
| *<I*/σ(*I*)> | 10.5 (1.3) |
| CC_1/2_ | 0.997 (0.553) |
| Refinement statistics |  |
| *R*_work_/*R*_free_ | 22.3/28.0 |
| Mean protein/solvent isotropic *B* factor (Å^2^) | 131.4/91.12 |
| Protein assembly in asymmetric unit (AU) | 1 monomer |
| Protein residues coded for in gene | 305 |
| Total modelled residues in AU |  |
| - protein residues by chain | 7-305 |
| - added waters | 6 |
| Matthews coefficient (Å^3^/Da) | 2.97 |
| Solvent content (%) | 58.6 |
| Ramachandran favoured/allowed/outliers (%) | 92.6/7.1/0.3 |
| RMSD bond lengths (Å) | 0.001 |
| RMSD bond angles (°) | 0.332 |
| PDB ID | 8BVI |
